# Application of CRISPR/Cas9 Tools for Genome Editing in the White-Rot Fungus *Dichomitus squalens*

**DOI:** 10.3390/biom11101526

**Published:** 2021-10-15

**Authors:** Joanna E. Kowalczyk, Shreya Saha, Miia R. Mäkelä

**Affiliations:** Department of Microbiology, University of Helsinki, Viikinkaari 9, 00790 Helsinki, Finland; joanna.ewa.kowalczyk@outlook.com (J.E.K.); shreya.suc@gmail.com (S.S.)

**Keywords:** *Dichomitus squalens*, CRISPR/Cas9, genome editing, ribonucleoprotein, single-stranded oligonucleotides

## Abstract

*Dichomitus squalens* is an emerging reference species that can be used to investigate white-rot fungal plant biomass degradation, as it has flexible physiology to utilize different types of biomass as sources of carbon and energy. Recent comparative (post-) genomic studies on *D. squalens* resulted in an increasingly detailed knowledge of the genes and enzymes involved in the lignocellulose breakdown in this fungus and showed a complex transcriptional response in the presence of lignocellulose-derived compounds. To fully utilize this increasing amount of data, efficient and reliable genetic manipulation tools are needed, e.g., to characterize the function of certain proteins in vivo and facilitate the construction of strains with enhanced lignocellulolytic capabilities. However, precise genome alterations are often very difficult in wild-type basidiomycetes partially due to extremely low frequencies of homology directed recombination (HDR) and limited availability of selectable markers. To overcome these obstacles, we assessed various Cas9-single guide RNA (sgRNA) ribonucleoprotein (RNP) -based strategies for selectable homology and non-homologous end joining (NHEJ) -based gene editing in *D. squalens*. We also showed an induction of HDR-based genetic modifications by using single-stranded oligodeoxynucleotides (ssODNs) in a basidiomycete fungus for the first time. This paper provides directions for the application of targeted CRISPR/Cas9-based genome editing in *D. squalens* and other wild-type (basidiomycete) fungi.

## 1. Introduction

Plant biomass degrading filamentous fungi are essential nutrient cyclers in terrestrial environments and important producers of enzymes and metabolites, for example, in different industrial sectors [1]. Due to their ecological and societal relevance, the number of whole genome-sequenced fungal species and strains is exponentially increasing together with post-genomic studies [2]. To effectively utilize these data to further understand fundamental physiological processes in fungi and to expand their use in biotechnology, precise and versatile methods for manipulation of fungal genomes are needed [3].

The discovery of CRISPR/Cas9 (clustered regularly interspaced short palindromic repeats/CRISPR-associated protein-9 nuclease form *Streptococcus pyogenes*) and its application to genome engineering [4], have revolutionized research on several scientific areas in less than a decade. The success of this technology is based on the ability of the Cas9 nuclease to recognize and cut a specific DNA sequence in the genome. This specificity is ensured by a single RNA molecule (i.e., guide RNA) that can be customized to target any genomic location in proximity to the protospacer adjacent motif (PAM). To survive the resulting double strand breaks (DSBs) the edited organism must alter its DNA so that it is no longer recognizable by the CRISPR-associated nuclease. This is mediated by the endogenous DSB repair systems.

In filamentous fungi, DSBs are dominantly repaired by error-prone, non-homologous end joining (NHEJ) [5,6,7] that often leads to indels and frame shifts within the target sequence. When a DNA sequence with homology close to the DSB (a so-called donor DNA, dDNA) is available, DSB can also be corrected through homologous recombination, often called homology directed recombination (HDR). HDR-mediated recombination allows for precise gene knockouts/knock-ins and is generally more predictable, and thus better suited, for targeted genome engineering. However, the frequency of HDR events is critically low in filamentous fungi leaving the process of targeted integration of DNA to be very inefficient [8]. Deletion of genes involved in NHEJ pathways, such as the *ku70*/*ku80* gene, has been shown to result in high HDR frequency in both filamentous ascomycete and basidiomycete fungi [5,9,10,11,12]. Therefore NHEJ-deficient strains are often used as a background for genetic manipulations.

To date, CRISPR/Cas9-based editing tools have been adapted for a number of well-studied ascomycete and basidiomycete species [13,14,15,16]. The development of these tools has opened new possibilities, e.g., to accelerate the discovery of novel secondary metabolites through the characterization and activation of previously unstudied biosynthetic gene clusters [17], molecular breeding of cultivated mushrooms [14] and functional analysis of not only individual genes but also large gene families [18], just to mention few.

The described CRISPR/Cas9-based strategies in filamentous fungi include, e.g., genomic integration of Cas9 and single chimeric guide RNA (sgRNA) encoding sequences [7,16,19,20], transient expression of Cas9 and sgRNA from a non-replicating plasmid [13,14,21,22], in vitro expression of guide RNA (gRNA) [15,23], expression of Cas9 from a self-replicating plasmid containing AMA1 (autonomous maintenance in *Aspergillus*) sequence [16] or *Ustilago maydis* ARS element [24] and delivery of in vitro assembled ribonucleoprotein (RNP) complexes that consist of Cas9 and gRNA [15]. However, in vivo Cas9 expression has been shown to cause unwanted phenotypes, such as delayed growth [25], further rearrangements or off-target mutations [26] and even toxic effects in host cells [27,28]. Transformed plasmids could also be degraded by endogenous nucleases into small DNA fragments, which may increase unwanted on- and off-target insertions in host cells [29]. In contrast, RNP-based genome editing is not limited by the efficiency of Cas9 and gRNA expression in vivo and it may protect gRNA from degradation [30]. As the RNPs are rapidly degraded after the transient exposure of the cells to Cas9, the chance of further rearrangements or off-target events that lead to unintended and nonspecific mutations is also lower [15].

Despite these recent developments, genetic tools for most filamentous fungal species are still poorly established or unavailable. For instance, there is little work showing effective strategies to induce HDR when NHEJ-deficient strains are not available. This is particularly true for wood-degrading, polyporous basidiomycete fungi that hold a largely untapped potential for plant biomass-related applications [31]. In this study, we assessed different methods for CRISPR/Cas9-based gene editing in the wild-type *Dichomitus squalens* strain. *D. squalens* is a transformable, wood-degrading fungus and a highly suitable reference species to investigate molecular mechanisms behind plant biomass degradation in white-rot basidiomycetes [32,33,34]. Here, we adapted a co-targeting strategy based on pre-assembled Cas9-sgRNA RNPs for selectable homology and NHEJ-based gene editing in *D. squalens*. To our knowledge, this is the first report on a successful induction of HDR-based genetic modifications using single-stranded oligodeoxynucleotides (ssODNs) in a basidiomycete fungus.

## 2. Materials and Methods

### 2.1. Strains and Growth Conditions

The wild-type (WT), monokaryotic *D. squalens* strain CBS464.89, derived from the WT dikaryon *D. squalens* FBCC312, was obtained from the FBCC-HAMBI culture collection (www.helsinki.fi/hambi/ accessed on 01 March 2018) and maintained at 28 °C on 2% (*w*/*v*) malt extract, 1.5% (*w*/*v*) agar (MEA) plates. The cultures for protoplast preparation were performed as previously described [33]. Transformants were selected on regeneration agar with 18–25 µg/mL geneticin (G-418, Roche, Mannheim, Germany) or 0.5–2 µg/mL carboxin (Sigma-Aldrich, St. Louis, MO, USA) or 1–2 mg/mL 5-fluoroorotic acid (5-FOA, Thermo Scientific, Vilnus, Lithuania) and 20 mM uridine (Molekula, Darlington, UK). The positive transformants were subcultured on MEA plates containing selective pressure and then maintained on MEA. For growth assays, mycelia-covered plugs (0.5 cm in diameter) from a freshly grown MEA plate were used to inoculate a low-nitrogen, asparagine-succinate (LN-AS, pH 4.5) medium with 1.5% (*w*/*v*) agar [35] and 25 mM of glucose, xylose, arabinose or galactose as a carbon source. Media of the uridine auxotrophic mutants were supplemented with 20 mM uridine. The strains used in this study are listed in Table 1.

### 2.2. Construction of Synthetic Guide RNA

All genetic modifications were designed with *D. squalens* CBS464.89 v1.0 genome and annotation (https://mycocosm.jgi.doe.gov accessed on 15 July 2018). The CRISPR sites (i.e., the target-specific CRISPR RNA or crRNA sequences) were identified using the Geneious R11 v11.1.4 software [37] and assessed based on their location (exons preferred), on-target activity [38] and off-target specificity score [39]. Since the cleavage efficiency of a CRISPR sequence at its target depends on many factors and is not yet well understood, one to three sequences per gene were selected for gRNA synthesis (Table S1). The guide RNAs (gRNAs), each containing a target complimentary crRNA and an auxiliary trans-activating crispr RNA (tracrRNA), were in vitro transcribed using the GeneArt™ Precision gRNA Synthesis kit (Thermo Scientific) and custom primers (Table S2) according to the manufacturer’s instructions.

### 2.3. Assembly of Cas9-gRNA Ribonucleoproteins and D. squalens Transformation

For formation of ribonuceoprotein (RNP) complexes, a commercial Cas9 nuclease containing a nuclear localization signal (Cas9-NLS; Thermo Scientific) was mixed with an in vitro synthesized gRNA in equimolar concentrations and a total volume of 10 µL in nuclease-free water. The Cas9-gRNA RNPs were formed after 10 min of incubation in RT and were stable in RT for ~1 h. The functionality of RNPs was tested in vitro by mixing ~900 ng of PCR-amplified DNA fragment (~1 kb) surrounding the CRISPR/Cas9 cut site with pre-assembled RNP complexes and incubating for 30 min at 37 °C. To digest Cas9, 1 U of Proteinase K was added and the reaction was incubated for 15 min at RT. Samples were visualized on 1% agarose gel.

For the in vitro RNP-mediated gene editing, 3 µg Cas9 and 600 ng gRNA were complexed into RNPs and transformed to ~2 million *D. squalens* protoplasts in STC (1.33 M sorbitol, 10 mM Tris-HCl of pH 7.5, 50 mM CaCl_2_). To induce homologous recombination, a repair template, i.e., the donor DNA (dDNA), was co-transformed with RNPs at concentrations indicated below. The PEG-mediated transformation was performed as previously described [33], except that aurintricarboxylic acid ammonium salt (ATA) was omitted in the RNP experiments as it has been shown to lead to inactivation of Cas9 [15].

### 2.4. Construction of Donor DNA

Two classical homology-based repair temples, i.e., dDNA with antibioticres selection marker surrounded by the flanking regions of a gene of interest, were constructed for deletion of the *D. squalens ku80* gene (protein ID: 828988). First, the ~1.7 kb upstream and downstream *ku80* flanking regions were amplified with Phusion™ High Fidelity DNA polymerase (Thermo Scientific, Vilnus, Lithuania) using custom primers (Table S3) and *D. squalens* CBS464.89 gDNA as a template. The G-418 resistance cassette, containing the neomycin phosphotransferase II encoding gene *nptII* driven by the *Flammulina velutipes GPD* promoter, was amplified from the pFungiway8 plasmid [40] with primers containing appropriate overhangs. To assemble the linear *ku80* deletion cassette (~5 kb), the three fragments were connected by fusion PCR amplification with nested primers (Figure S1A). The split marker *ku80* deletion cassette was constructed by connecting the partial G-418 resistance cassette sequence with upstream (~2.9 kb) and downstream (~2.6 kb) flanking regions of the *ku80* gene (Figure S1B). The 448 bp overlap in the G-418 resistance cassette part of the sequence allows recombination of the split marker inside the cells. Approximately 10 µg of linear or split-marker dDNA was used for transformation with Cas9-gRNA RNPs.

For construction of a ~2 kb microhomology cassette, G-418 resistance cassette was amplified from pFungiway8 plasmid with custom primers carrying 40 bp long microhomology arms complementary to the flanking regions of the *ku80* gene (Table S3, Figure S1C). Approximately 10 µg of microhomology dDNA was co-transformed with dual Cas9-gRNA RNPs for gene deletion in WT *D. squalens*.

Custom ssODNs were synthesized (Eurofins Genomics, Konstanz, Germany) and used as dDNA-inducing single nucleotide changes in the target loci (Table S4). The nucleotide changes were designed to (a) cause missense or nonsense mutations that lead to truncation of targeted protein, (b) introduce or mutate restriction sites for rapid verification of edited strains and (c) alter the PAM to prevent Cas9 from re-cutting the target sequence once the desired edit has been introduced at the locus. A total of 5–10 µg of ssODNs were co-transformed with Cas9-gRNA RNPs for a single gene knockout in WT *D. squalens*.

### 2.5. Analysis of D. squalens Mutants

To verify edits at the targeted locus, genomic DNA was extracted from randomly selected *D. squalens* transformants with extraction buffer (2% N-cetyl-N,N,Ntrimethylammonium bromide (CTAB), 100 mM Tris-HCl, 1.4 M NaCl, 20 mM EDTA and 0.2% β-mercaptoethanol) and purified with chloroform-isoamyl alcohol (24:1) according to Chang et al. [41] and quantified by NanoDrop™ One Microvolume UV-Vis Spectrophotometer (Thermo Scientific).

The ~1000 bp long DNA region surrounding the Cas9 cut was amplified by a PCR with GoTag^®^ Green Master Mix (Promega, Madison, WI, USA) and custom primers (Table S5), according to the manufacturer’s instructions. For strains with mutated or inserted restriction sites, the amplicons were digested with FastDigest enzymes (Thermo Scientific, Vilnus, Lithuania) according to the manufacturer’s instructions and visualized on 1% agarose gel. Mutants with desired restriction pattern were amplified again, this time using Phusion™ High-Fidelity DNA polymerase (Thermo Scientific, Vilnus, Lithuania), and the PCR products were Sanger sequenced (Macrogen, Amsterdam, The Netherlands) to confirm edits at the locus. The generated chromatograms were manually proofread using Chromas v. 2.4.4 software.

To confirm the stability of the CRISPR-created edits, the mutant strains we repeatedly cultivated over four generations on 2% MEA plates, after which DNA was extracted and amplified with custom primers as described above. The PCR products from the fourth generation strains were Sanger sequenced to assess the stability of the edits.

## 3. Results and Discussion

### 3.1. RNPs Are Functional In Vivo and Introduce Double Strand DNA Breaks

**Figure 1:**
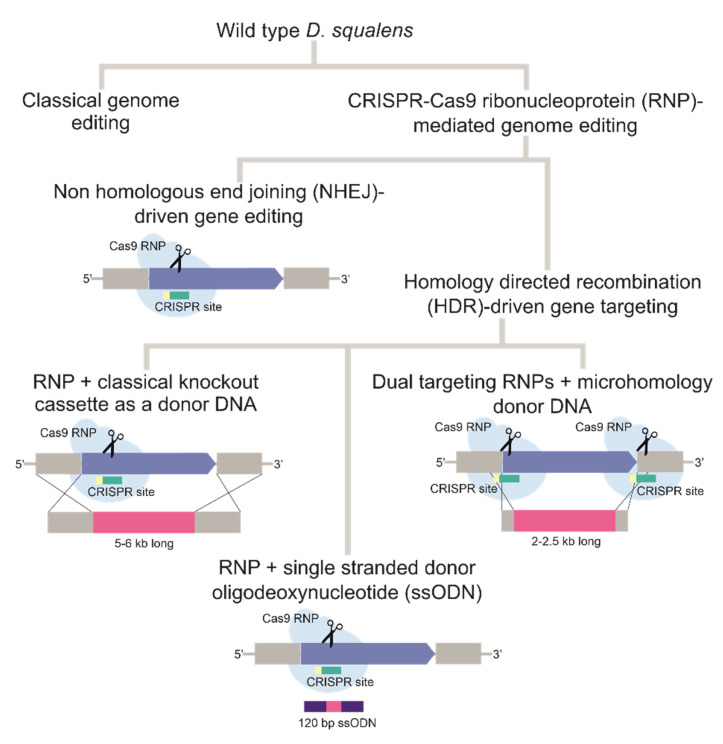
Flow diagram of the different approaches in the wild-type *D. squalens* strain.

In this work we evaluated different methods for CRISPR/Cas9-based gene editing in the WT *D. squalens* (Figure 1). First, we tested whether Cas9 could be delivered into *D. squalens* protoplasts using PEG-mediated transformation for introduction of double strand breaks (DBS) in the target sequences determined by in vitro synthesized gRNAs. The orotidine 5′-phosphate decarboxylase (OMP decarboxylase, EC 4.1.1.23), encoded in fungi by the *ura3*/*pyrG* gene, catalyzes the conversion of orotidine 5′-phosphate to uridine 5′-phosphate in the *de novo* pyrimidine biosynthesis pathway [42]. Inactivation of *ura3*/*pyrG* leads to easily distinguishable phenotypes (uridine/uracil auxotrophy and 5-FOA resistance) and provides a well-selectable marker for further gene editing. Therefore, we chose to target this gene with the CRISPR/Cas9 system in WT *D. squalens*. The OMP decarboxylase orthologue in *D. squalens* (*ura3*, protein ID: 954994) was identified from a bi-directional protein sequence homology search using previously characterized *pyrG*/*URA3* genes from the white-rot fungi *Pleurotus ostreatus* [43] and *Ganoderma lucidum* [44] as a query.

**Figure 2:**
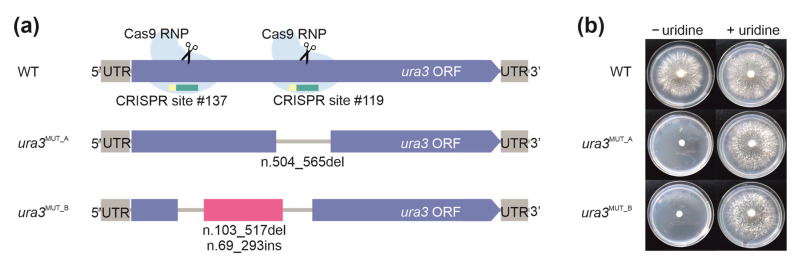
CRISPR/Cas9 RNPs were used for targeted mutagenesis of the *ura3* gene, encoding orotidine 5′-phosphate decarboxylase, in the wild-type *D. squalens* strain (WT): (**a**) schematic representation of the Cas9 target sites in the WT *ura3* gene and mutations introduced by NHEJ to *ura3*^MUT_A^ and *ura3*^MUT_B^ strains and (**b**) the *ura3* mutants have defects in the pyrimidine biosynthesis pathway and require exogenous uridine supplementation.

The pre-assembled RNP mixtures targeting the first, second or third exon of *ura3* were transformed independently (a single RNP per transformation) and collectively (dual RNPs per transformation) into WT *D. squalens* protoplasts. This resulted in >100 colonies, including two fast growing transformants with strong resistance to 5-FOA and uridine auxotrophy. The knockout mutants, named *ura3*^MUT_A^ and *ura3*^MUT_B^, originated from a transformation in which both the first and third exon of the *ura3* gene were targeted simultaneously (Figure 2). Sanger sequencing confirmed that both mutants had insertions and deletions (indels) in the *ura3* sequence near the programmed Cas9 cuts (Figure 2) that are characteristic to the error-prone DSB repair by NHEJ. Additionally, sequencing unraveled that the *ura3*^MUT_A^ had a 61 bp deletion near CRISPR site #119 (Figure S2A), suggesting that only one RNP was delivered to the nucleus during transformation. Efficiency of RNP delivery into fungal protoplasts with subsequent translocation to the nucleus is challenging and it has previously been reported to result in low genome editing efficiency in the ascomycete species *Penicillium chrysogenum* [15] and *Trichoderma reesei* [30]. The addition of surfactants is one option to improve cell membrane permeability during transformation, and Triton X-100 was shown to significantly increase the efficiency of RNP delivery in PEG-mediated transformation in *T. reesei* [30]. Furthermore, NHEJ has the disadvantage of introducing random insertions and translocations that affect neighboring genes [12]. We also observed this as the second mutant, *ura3*^MUT_B^, had a 414 bp long deletion between CRISPR sites #137 and #119 and an additional 294 bp insertion (Figure S2B) that was identified as a fragment of a neighboring gene (protein ID: 915108) that was annotated as a putative meiotic cell division protein.

Based on these results, we concluded that although RNPs introduced DSBs by the Cas9 nuclease at the expected site, which lead to loss-of-function mutations when repaired by NHEJ in WT *D. squalens*, the extremely low frequency of NHEJ (<1% gene targeting efficiency) prevents it from being a useful method in the absence of an easily identifiable phenotype. This is in line with the previous observation on the low efficiency of NHEJ-directed repair in the basidiomycete fungus *Schizophyllum commune* after introduction of CRISPR/Cas9-mediated DSBs [45].

We did not observe any *D. squalens* colonies with spontaneous uridine auxotrophy appearing without CRISPR/Cas9-mediated mutagenesis at the *ura3* locus. Among the remaining slow growing 5-FOA resistant transformants, few randomly selected colonies were still able to grow on LN-AS minimal medium plates without uridine (data not shown), thus indicating leaky selection when 1 mg/mL 5-FOA is used. To increase selectivity, we recommend using higher concentrations of 5-FOA and, e.g., 2 mg/mL 5-FOA seem to inhibit growth of WT *D. squalens* mycelium (data not shown).

### 3.2. Co-Transformation of a Long Homology-Based Repair Cassette Does Not Induce HDR in WT D. squalens

Next, we tested whether gene deletions could be created in WT *D. squalens* via HDR. With the general preference for NHEJ over the HDR DNA repair system, reaching workable levels of HDR is a major challenge in higher fungi. The Ku70/Ku80 protein complex is involved in the NHEJ repair pathway, and one of the common approaches to enrich HDR in filamentous fungi is the construction of *ku70*/*ku80* mutants in which NHEJ is reduced [6,10,12]. However, the addition of dDNA with a certain amount of homology to CRISPR/Cas9 experiments has been shown to induce HDR-based mutations in asco- and basidiomycete fungi without the need for construction of NHEJ-deficient mutants [45,46,47].

To study whether the addition of dDNA to RNP transformation could induce HDR-driven mutations in WT *D. squalens*, we chose to target its *ku80* orthologue with pre-assembled Cas9 RNPs. The orthologue of the *ku80* gene, encoding a putative protein involved in NHEJ (*ku80*, protein ID: 828988), was identified from the *D. squalens* genome with a bi-directional protein sequence homology search using the Ku80 sequence from the *S. commune* genome [10] as a query. We constructed and tested two classical homology-based gene deletion dDNA cassettes, linear and split marker, with ~1.6 kb *ku80* flanking regions surrounding the *nptII* selection marker conferring geneticin resistance (Figure S1). Despite several attempts with both linear and split cassettes, we were not able to obtain transformants in which a Cas9-introduced cut at the *ku80* locus would have been repaired by the incorporation of the supplied template. Since the delivery of Cas9 to the nucleus was not 100% effective in our previous experiment, in which *ura3* was targeted with the pre-assembled RNP mixtures, it is likely that the delivery of 3–5 kb long dDNA constructs could be similarly problematic.

In an attempt to decrease the donor size, we constructed a microhomology repair template [46], with *nptII* selection marker flanked by ~40 bp long DNA sequences homologous to *ku80* flanking regions, and co-transformed it to WT *D. squalens* with two RNPs targeting Cas9 to the ends of the *ku80* gene (Figure S1C). Despite the dDNA size of <2 kb, and although in several ascomycete species flanking regions of 30–60 bp have been shown to induce HDR in CRISPR/Cas9 systems [15,16,20], we did not obtain any geneticin resistant colonies using this method. From these results, we concluded that the presence of a long dDNA does not increase HDR rates in WT *D. squalens*.

### 3.3. Presence of a Short Oligonucleotide Repair Template Induce HDR-Based Gene Editing up to 60%

In order to investigate if co-transformation of shorter repair templates induces HDR-driven events in WT *D. squalens*, we designed 60–120 bp long single-stranded oligonucleotide (ssODN) donors, which were delivered to protoplasts with the pre-assembled RNP complexes. With this approach, we aimed to create nucleotide changes in an iron–sulphur protein subunit of succinate dehydrogenase encoding gene *sdi1*. Previous studies showed that the substitution of a histidine residue for leucine within the third cysteine-rich cluster of that gene confers resistance to fungicide carboxin in *U. maydis* [48]. The succinate dehydrogenase orthologue in *D. squalens* (*sdi1*, protein ID: 927354) was identified from a bi-directional protein sequence homology search using Sdi1 sequences from the basidiomycete fungi *Lentinula edodes* [49] and *P. ostreatus* [50] along with the ascomycete species *Magnaporthe oryzae* [47] as a query. The alignment of Sdi1 amino acid sequences from these species showed that the histidine residue that was targeted for carboxin resistance is conserved among these species, thus making it a strong candidate for a point mutation.

**Figure 3:**
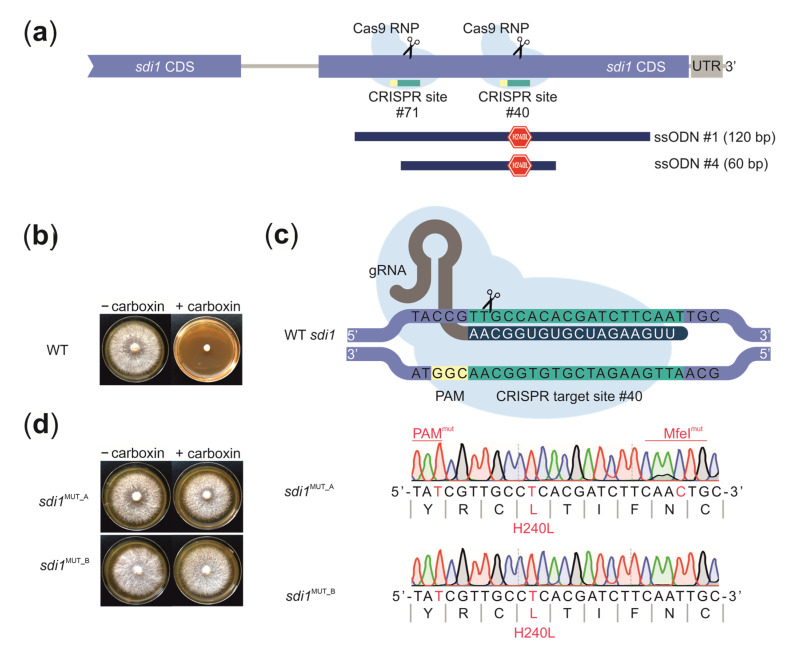
(**a**) Two ssODN donors with varying length were designed to create a missense mutation H240L in the last exon of the *D. squalens sdi1* gene. (**b**) The wild-type *D. squalens* (WT) is sensitive to fungicide carboxin. (**c**) Schematic representation of the Cas9 target site in the *D. squalens sdi1* gene; the missense and nonsense mutations introduced by Cas9-mediated homologous recombination at the *sdi1* locus are shown. (**d**) The *sdi1*^H240L^ mutants are carboxin resistant.

The ssODN donors with varying length were designed to create a missense mutation H240L in *D. squalens sdi1* and two silent mutations in the protospacer-adjacent motif (PAM) and MfeI restriction site to facilitate the verification of transformants (Figure 3A). WT *D. squalens* protoplasts were co-transformed with in vitro assembled RNPs and 5 µg of ssODNs and selected on carboxin containing medium. Resistant colonies appeared after six days of growth, confirming that *sdi1* editing leads to carboxin resistance in *D. squalens*. The majority of resistant colonies (28) resulted from a transformation in which gRNA directing Cas9 near the targeted histidine residue (CRISPR site #40) and 120 bp long ssODN #1 donor were present (Figure 3). A much lower number of resistant colonies appeared when the same CRISPR site was targeted with 60 bp long ssODN #4 donor (two colonies) and when the more distant CRISPR site #71 was targeted with 120 bp long donor (one colony). This not only suggests that the length of the ssODN but also the distance between the modification and the cut site are important factors that need to be optimized for efficient editing events.

Restriction digestion assay indicated the introduction of the desired sequence changes at the *sdi1* locus in ~55% transformants (17/31). This was further confirmed by Sanger sequencing of the restriction analysis based on five positive and one negative transformant, all of which were shown to have identical H240L missense mutation introduced by RNP-based targeted mutagenesis via HDR (Figure 3C). Accordingly, the desired mutation in the MfeI restriction site was present in five out of six sequenced *sdi1* mutants, indicating that the actual incidence of HDR-based gene edits is close to 60% or higher. To our knowledge, this is a first report of ssODN being successfully used to induce HDR-based genetic modifications in a basidiomycete fungus. Previously, ssODNs have been shown to mediate highly efficient CRISPR gene editing in the ascomycetous *Aspergillus* species when transformed together with a self-replicating plasmid expressing Cas9 and sgRNAs [51].

Interestingly, four out of six transformants had the designed mutation in the neighboring PAM and only one transformant in the further located PAM sequence. This suggests that the mutation frequency strongly decreases when the distance between the modification and the cut site is more than 40 bp. Additionally, we did not observe carboxin-resistant *D. squalens* colonies appearing from a transformation in which Cas9 RNPs were used without the addition of ssODNs, i.e., from NHEJ-based DNA repair events, confirming our observations on extremely low incidence of not only HDR but also NHEJ-based repair events in WT *D. squalens*.

### 3.4. Coediting Allows Selection of Mutants with Edits at the Gene of Interest in the Wild-Type D. squalens

**Figure 4:**
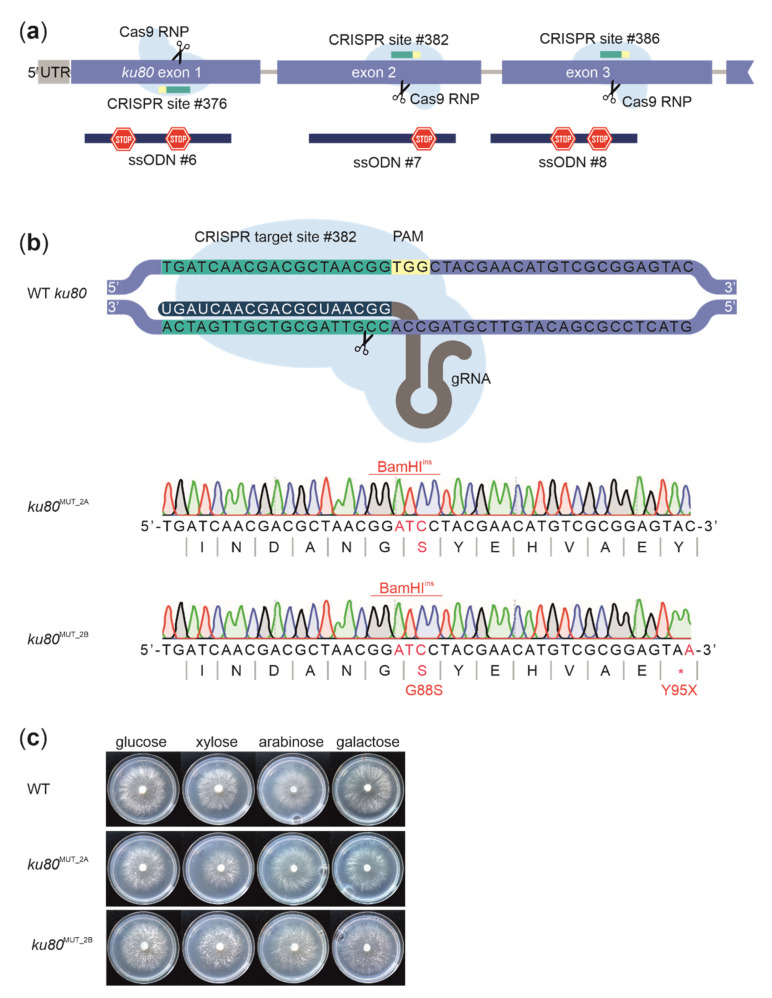
(**a**) The first, second or third exon of the *D. squalens ku80* gene were targeted for introduction of STOP codon(s) (*). (**b**) Schematic representation of the Cas9 target site in the second exon of the *ku80* gene of the wild-type *D. squalens* (WT); obtained edits in *ku80*^MUT_2A^ and *ku80*^MUT_2B^ strains are shown. (**c**) The *ku80*^MUT_2A^ and *ku80*^MUT_2B^ mutants are phenotypically indistinguishable from the WT.

To be able to simultaneously edit two loci using ssODN donors, we tested whether selection for *sdi1*^H240L^ mutants could result in identification of HDR-based mutations at a second locus in WT *D. squalens*. Coediting with ssODNs has been shown to be functional, e.g., in mammalian cells [52] and some ascomycetes [47,53]. For the second target, we chose to knockout the *ku80* gene. For that we introduced two STOP codons in the first and the third exon and one STOP codon on the second exon of this gene (Figure 4A). Protoplasts were transformed with RNPs targeting both *sdi1* and *ku80,* along with respective 120 bp long donor ssODNs, and selected with carboxin. In total 10 µg of ssODNs were used, corresponding to 5 µg for repair at both loci. Restriction digests indicated that ~20% of the carboxin-resistant transformants had nonsense mutations at the *ku80* locus in one of the targeted exons. Sanger sequencing further confirmed that the desired edits were introduced in five out of six positively identified mutants. This demonstrated that coediting can be successfully used to enrich for mutations at the second locus in WT *D. squalens* and it also enables identification of transformants based on the marker that has been mutated alongside the target loci.

Similarly to previous results with single locus targeting, the mutations closer to the predicted Cas9 cut had higher frequency of being incorporated to the genome. For example, during editing of the second exon of *ku80*, the mutations leading to incorporation of the BamHI restriction site at the PAM sequence were more common than the nonsense mutation Y95X, designed 27 bp from the Cas9 cut site (Figure 4B). Among the sequenced *D. squalens* mutant strains, five had nonsense mutations in the coding sequence of the *ku80* gene that were designed to truncate the encoded protein. These included two strains (*ku80*^MUT_1A^ and *ku80*^MUT_1B^) with the S21X mutation in the first exon, one strain (*ku80*^MUT_2B^) with the Y95X mutation in the second exon and two strains (*ku80*^MUT_3A^ and *ku80*^MUT_3B^) with the Y135X or Y135X-W142X mutation in the third exon (Table 1). The *ku80* mutants were not observed to have growth defects and were otherwise phenotypically indistinguishable from the WT strain (Figure 4C and data not shown).

**Figure 5:**
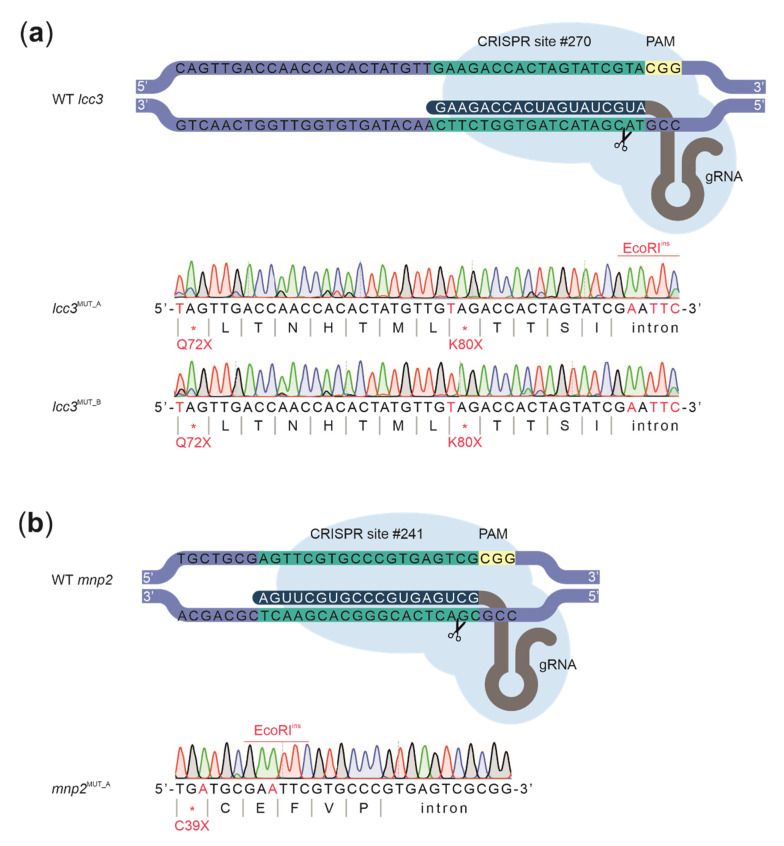
Schematic representation of the Cas9 target site in the *lcc3* and the *mnp2* gene of the wild type *D. squalens* (WT): (**a**) two knockout mutants, *lcc3*^MUT_A^ and *lcc3*^MUT_B^, with the shown edits were obtained and (**b**) for the *mnp2*, a knockout mutant *mnp2*^MUT_A^ with the shown edits was obtained. * = STOP codon.

To overcome the cut-to-mutation distance correlation, we increased the amount of the donor ssODNs supplied during transformation. For that, we coedited *sdi1* along with a laccase encoding gene *lcc3* (protein ID: 59186). Laccases are multi-copper oxidases that have various suggested biological roles including participation in lignocellulose degradation [31]. Due to their broad substrate specificity, laccases also are attractive biocatalysts for a wide range of biotechnology applications. The previous studies have shown that *lcc3* is highly expressed by *D. squalens* in various cultivation conditions [32,34,54], which made it an interesting target to study its role in vivo in more detail. The protoplast mixture was supplied with 7 and 10 µg of ssODNs for the repair of cuts introduced in the *sdi1* and *lcc3* loci, respectively, while the total amount of Cas9 and gRNA used to assemble RNPs remained unchanged. In this case, we chose to use a higher amount of dDNA for *lcc3* to enrich for edits at this locus among carboxin-resistant colonies. Out of 10 carboxin-resistant transformants, two showed the restriction pattern correlating with EcoRI incorporation at the *lcc3* locus, which was later confirmed by Sanger sequencing (Figure 5A). Interestingly, both *lcc3*^MUT_A^ and *lcc3*^MUT_B^ knockouts had the desired nonsense mutations Q72X and K80X located 40 and 16 bp from the predicted Cas9 cut, respectively. This suggests that the amount of ssODNs could be an important factor when dealing with increasing cut-to-mutation distances during HDR-mediated gene editing with pre-assembled RNPs.

We further confirmed these results by using the same set-up for the coediting of *sdi1* with the *mnp2* gene (protein ID: 578774). Manganese peroxidases (MnPs) are key enzymes for lignin degradation and uniquely produced by white-rot fungi [31]. As the *D. squalens* MnP2 encoding gene has been reported to be highly expressed in several wood and other plant biomass containing cultures [32,34,54,55], we were interested to target it to obtain a strain that possibly has an altered ability for lignocellulose conversion. In this case, one out of 10 carboxin-resistant transformants showed the expected EcoRI restriction pattern. Sanger sequencing revealed that the *mnp2*^MUT_A^ had a nonsense mutation C39X introduced 22 bp from the predicted Cas9 cut (Figure 5B). Furthermore, all CRISPR-created edits, which we obtained by using ssODN repair templates, were stable over four generations of the mutant strains as confirmed by Sanger sequencing (data not shown).

While repair accuracy for edits at increased cut-to-mutation distance increased with higher amounts of ssODNs in the coediting experiments, we did not observe higher targeting efficiencies for *lcc3* and *mnp2*. Although careful optimization of sgRNAs and ssODNs is necessary, this could also be linked to the accessibility of the targeted genes/regions in the chromatin as we observed mutation efficiencies at the second locus between 10 and 60%, when other genes were coedited in the WT *D. squalens* (data not shown). For example, regions of low transcriptional activity have been suggested to complicate the direction of Cas9 by sgRNA [23].

Our study demonstrated that precise genome alterations in *D. squalens* could be obtained by CRISPR/Cas9-based RNP-mediated editing. To our knowledge, induction of HDR-driven genetic modifications by ssODNs in a basidiomycete species has not been previously reported. We showed that shorter dDNA (e.g., 120 bp ssODN) co-transformed with RNPs induced HDR-mediated gene editing with the efficiency of up to 60% at a single locus and 20% at dual loci in WT *D. squalens*, which indicates that the method presented here provides an opportunity to develop a genome editing system in non-reference filamentous fungal species, e.g., without available NHEJ-deficient strains. In addition, the chemical synthesis of these relatively short oligonucleotide templates can be considered cost effective, supporting the feasibility of this approach. The future work will concentrate on further optimization of the coediting strategy presented here to increase its efficiency, which could allow, e.g., multiplexing of several loci. In addition, the NHEJ-deficient *D. squalens ku80* knockout strains obtained here provide opportunities for their use as parental strains for genetic modifications with likely improved HDR frequency.

## 4. Conclusions

In this study we showed that CRISPR/Cas9-based editing with pre-assembled Cas9-sgRNA RNPs enables precise genome alterations in the basidiomycete white-rot fungus *D. squalens*, for which NHEJ-deficient strains were not available before. We also presented a successful induction of HDR-based genetic modifications by using ssODNs for the first time in a basidiomycete fungus, and demonstrated successful coediting of two loci using ssODN donors. This opens up new possibilities to study gene function and develop improved strains for biotechnology applications in *D. squalens* that is an interesting reference species for white-rot wood degradation. In addition, the methods presented here can most likely be adapted to, e.g., other plant biomass degrading basidiomycete fungi, for which very limited tool sets are often available for genetic modifications.

## Figures and Tables

**Table 1:** *Dichomitus squalens* strains used in this study.

*D. squalens* Strains	Description	Genotype	Reference
CBS464.89 (WT)	Wild-type (WT)-derived monokaryon.		[36]
CBS464.89 *ura3*^MUT_A^	Uridine auxotroph.	*ura3* n.504_565del in WT.	This study.
CBS464.89 *ura3*^MUT_B^	Uridine auxotroph.	*ura3* n.103_517del in protein ID: 915108 and n.69_363ins in WT.	This study.
CBS464.89 *sdi1*^MUT_A^	Carboxin-resistant strain.	*sdi1* p.H240L in WT.	This study.
CBS464.89 *sdi1*^MUT_B^	Carboxin-resistant strain.	*sdi1* p.H240L in WT.	This study.
CBS464.89 *ku80*^MUT_2A^	Carboxin-resistant strain with missense mutation in *ku80*.	*sdi1* p.H240L, *ku80* p.G88S in WT.	This study.
CBS464.89 *ku80*^MUT_2B^	Carboxin-resistant and NHEJ-deficient strain.	*sdi1* p.H240L, *ku80* p.G88S and p.Y95X in WT.	This study.
CBS464.89 *ku80*^MUT_1A^	Carboxin-resistant and NHEJ-deficient strain.	*sdi1* p.H240L, *ku80* p.P19S and p.S21X in WT.	This study.
CBS464.89 *ku80*^MUT_1B^	Carboxin-resistant and NHEJ-deficient strain.	*sdi1* p.H240L, *ku80* p.V16F, p.P19S and p.S21X in WT.	This study.
CBS464.89 *ku80*^MUT_3A^	Carboxin-resistant and NHEJ-deficient strain.	*sdi1* p.H240L, *ku80* p.Y135X in WT.	This study.
CBS464.89 *ku80*^MUT_3B^	Carboxin-resistant and NHEJ-deficient strain.	*sdi1* p.H240L, *ku80* p.Y135X and W142X in WT.	This study.
CBS464.89 *lcc3*^MUT_A^	Carboxin-resistant strain with *lcc3* knockout.	*sdi1* p.H240L, *lcc3* p.Q72X and p.K80X in WT.	This study.
CBS464.89 *lcc3*^MUT_B^	Carboxin-resistant strain with *lcc3* knockout.	*sdi1* p.H240L, *lcc3* p.Q72X and p.K80X in WT.	This study.
CBS464.89 *mnp2*^MUT_A^	Carboxin-resistant strain with *mnp2* knockout.	*sdi1* p.H240L, *mnp2* p.C39X in WT.	This study.

## Data Availability

All data are contained within the article and Supplementary Materials.

## Supplementary Materials

The following are available online at https://www.mdpi.com/article/10.3390/biom11101526/s1. Figure S1: construction of HDR-based donor DNA for repair of DNA breaks introduced by gRNA/Cas9 RNPs at ku80 locus; Figure S2: Sanger sequencing of *D. squalens ura3*^MUT_A^ and *ura3*^MUT_B^ strains; Table S1: sequences of CRISPR/Cas9 guides; Table S2: PCR primers used for guide RNA synthesis; Table S3: primers used to construct homology-based repair temples for *ku80* deletion; Table S4: sequences of ssODNs; Table S5: primers used for verification of edited loci.

## References

[1] Meyer V., Basenko E.Y., Benz J.P., Braus G.H., Caddick M.X., Csukai M., de Vries R.P., Endy D., Frisvad J.C., Gunde-Cimerman N. (2020). Growing a circular economy with fungal biotechnology: A white paper. Fungal Biol. Biotechnol..

[2] de Vries R.P., Mäkelä M.R. (2020). Genomic and postgenomic diversity of fungal plant biomass degradation approaches. Trends Microbiol..

[3] Ouedraogo J.-P., Tsang A. (2020). CRISPR—Cas systems for fungal research. Fungal Biol. Rev..

[4] Doudna J.A., Charpentier E. (2014). The new frontier of genome engineering with CRISPR-Cas9. Science.

[5] Ninomiya Y., Suzuki K., Ishii C., Inoue H. (2004). Highly efficient gene replacements in *Neurospora* strains deficient for nonhomologous end-joining. Proc. Natl. Acad. Sci. USA.

[6] Kück U., Hoff B. (2010). New tools for the genetic manipulation of filamentous fungi. Appl. Microbiol. Biotechnol..

[7] Fuller K.K., Chen S., Loros J.J., Dunlap J.C. (2015). Development of the CRISPR/Cas9 system for targeted gene disruption in *Aspergillus fumigatus*. Eukaryot. Cell.

[8] Lichius A., Ruiz D.M., Zeilinger S., Nevalainen H. (2020). Genetic transformation of filamentous fungi: Achievements and challenges. Grand Challenges in Fungal Biotechnology.

[9] Aasland R., Stewart A.F., Gibson T., Abadio A.K., Kioshima E.S., Teixeira M.M., Martins N.F., Maigret B., Felipe M.S., Amselem J. (2011). Finished genome of the fungal wheat pathogen *Mycosphaerella graminicola* reveals dispensome structure, chromosome plasticity, and stealth pathogenesis. Mol. Microbiol..

[10] de Jong J.F., Ohm R.A., de Bekker C., Wösten H.A.B., Lugones L.G. (2010). Inactivation of Ku80 in the mushroom-forming fungus *Schizophyllum commune* increases the relative incidence of homologous recombination. FEMS Microbiol. Lett..

[11] Salame T.M., Knop D., Tal D., Levinson D., Yarden O., Hadar Y. (2012). Predominance of a versatile-peroxidase-encoding gene, *mnp4*, as demonstrated by gene replacement via a gene targeting system for *Pleurotus ostreatus*. Appl. Environ. Microbiol..

[12] Nakazawa T., Ando Y., Kitaaki K., Nakahori K., Kamada T. (2011). Efficient gene targeting in Δ*Cc.Ku70* or Δ*Cc.Lig4* mutants of the agaricomycete *Coprinopsis cinerea*. Fungal Genet. Biol..

[13] Wang P.-A., Xiao H., Zhong J.-J. (2020). CRISPR-Cas9 assisted functional gene editing in the mushroom *Ganoderma lucidum*. Appl. Microbiol. Biotechnol..

[14] Boontawon T., Nakazawa T., Inoue C., Osakabe K., Kawauchi M., Sakamoto M., Honda Y. (2021). Efficient genome editing with Crispr/Cas9 in *Pleurotus ostreatus*. AMB Express.

[15] Pohl C., Kiel J.A.K.W., Driessen A.J.M., Bovenberg R.A.L., Nygård Y. (2016). CRISPR/Cas9 based genome editing of *Penicillium chrysogenum*. ACS Synth. Biol..

[16] Nødvig C.S., Nielsen J.B., Kogle M.E., Mortensen U.H. (2015). A CRISPR-Cas9 system for genetic engineering of filamentous fungi. PLoS ONE.

[17] Jiang C., Lv G., Tu Y., Cheng X., Duan Y., Zeng B., He B. (2021). Applications of CRISPR/Cas9 in the synthesis of secondary metabolites in filamentous fungi. Front. Microbiol..

[18] van Leeuwe T.M., Arentshorst M., Ernst T., Alazi E., Punt P.J., Ram A.F.J. (2019). Efficient marker free CRISPR/Cas9 genome editing for functional analysis of gene families in filamentous fungi. Fungal Biol. Biotechnol..

[19] Katayama T., Tanaka Y., Okabe T., Nakamura H., Fujii W., Kitamoto K., Maruyama J.-I. (2016). Development of a genome editing technique using the CRISPR/Cas9 System in the industrial filamentous fungus *Aspergillus oryzae*. Biotechnol. Lett..

[20] Zhang C., Meng X., Wei X., Lu L. (2016). Highly efficient CRISPR mutagenesis by microhomology-mediated end joining in *Aspergillus fumigatus*. Fungal Genet. Biol..

[21] Arazoe T., Miyoshi K., Yamato T., Ogawa T., Ohsato S., Arie T., Kuwata S. (2015). Tailor-made CRISPR/Cas system for highly efficient targeted gene replacement in the rice blast fungus. Biotechnol. Bioeng..

[22] Matsu-ura T., Baek M., Kwon J., Hong C. (2015). Efficient gene editing in *Neurospora crassa* with CRISPR technology. Fungal Biol. Biotechnol..

[23] Liu R., Chen L., Jiang Y., Zhou Z., Zou G. (2015). Efficient genome editing in filamentous fungus *Trichoderma reesei* using the CRISPR/Cas9 system. Cell Discov..

[24] Schuster M., Schweizer G., Reissmann S., Kahmann R. (2016). Genome editing in *Ustilago maydis* using the CRISPR-Cas system. Fungal Genet. Biol..

[25] Enkler L., Richer D., Marchand A.L., Ferrandon D., Jossinet F. (2016). Genome engineering in the yeast pathogen *Candida glabrata* using the CRISPR-Cas9 system. Sci. Rep..

[26] Jacobs J.Z., Ciccaglione K.M., Tournier V., Zaratiegui M. (2014). Implementation of the CRISPR-Cas9 system in fission yeast. Nat. Commun..

[27] Cho J.S., Choi K.R., Prabowo C.P.S., Shin J.H., Yang D., Jang J., Lee S.Y. (2017). CRISPR/Cas9-coupled recombineering for metabolic engineering of *Corynebacterium glutamicum*. Metab. Eng..

[28] Jiang Y., Qian F., Yang J., Liu Y., Dong F., Xu C., Sun B., Chen B., Xu X., Li Y. (2017). CRISPR-Cpf1 Assisted Genome Editing of *Corynebacterium glutamicum*. Nat. Commun..

[29] Kim S., Kim D., Cho S.W., Kim J., Kim J.-S. (2014). Highly efficient RNA-guided genome editing in human cells via delivery of purified Cas9 ribonucleoproteins. Genome. Res..

[30] Zou G., Xiao M., Chai S., Zhu Z., Wang Y., Zhou Z. (2020). Efficient genome editing in filamentous fungi via an improved CRISPR-Cas9 ribonucleoprotein method facilitated by chemical reagents. Microb. Biotechnol..

[31] Mäkelä M.R., Hildén K., Kowalczyk J.E., Hatakka A., Nevalainen H. (2020). Progress and research needs of plant biomass degradation by basidiomycete fungi. Grand Challenges in Fungal Biotechnology.

[32] Rytioja J., Hildén K., Di Falco M., Zhou M., Aguilar-Pontes M.V., Sietiö O.-M., Tsang A., de Vries R.P., Mäkelä M.R. (2017). The molecular response of the white-rot fungus *Dichomitus squalens* to wood and non-woody biomass as examined by transcriptome and exoproteome analyses. Environ. Microbiol..

[33] Daly P., Slaghek G.G., Casado López S., Wiebenga A., Hilden K.S., de Vries R.P., Mäkelä M.R. (2017). Genetic transformation of the white-rot fungus *Dichomitus squalens* using a new commercial protoplasting cocktail. J. Microbiol. Methods.

[34] Kowalczyk J.E., Peng M., Pawlowski M., Lipzen A., Ng V., Singan V., Wang M., Grigoriev I.V., Mäkelä M.R. (2019). The white-rot basidiomycete *Dichomitus squalens* shows highly specific transcriptional response to lignocellulose-related aromatic compounds. Front. Bioeng. Biotechnol..

[35] Hatakka A.I., Uusi-Rauva A.K. (1983). Degradation of ^14^C-labelled poplar wood lignin by selected white-rot fungi. Eur. J. Appl. Microbiol. Biotechnol..

[36] Casado López S., Peng M., Daly P., Andreopoulos B., Pangilinan J., Lipzen A., Riley R., Ahrendt S., Ng V., Barry K. (2019). Draft genome sequences of three monokaryotic isolates of the white-rot basidiomycete fungus *Dichomitus squalens*. Microbiol. Resour. Announc..

[37] Kearse M., Moir R., Wilson A., Stones-Havas S., Cheung M., Sturrock S., Buxton S., Cooper A., Markowitz S., Duran C. (2012). Geneious basic: An integrated and extendable desktop software platform for the organization and analysis of sequence data. Bioinformatics.

[38] Doench J.G., Hartenian E., Graham D.B., Tothova Z., Hegde M., Smith I., Sullender M., Ebert B.L., Xavier R.J., Root D.E. (2014). Rational design of highly active sgRNAs for CRISPR-Cas9-mediated gene inactivation. Nat. Biotechnol..

[39] Hsu P.D., Scott D.A., Weinstein J.A., Ran F.A., Konermann S., Agarwala V., Li Y., Fine E.J., Wu X., Shalem O. (2013). DNA targeting specificity of RNA-guided Cas9 nucleases. Nat. Biotechnol..

[40] Nishikawa R., Yoshida M., Noda T., Okuhara T., Taguchi G., Inatomi S., Shimosaka M. (2016). pFungiway: A series of plasmid vectors used for gene manipulation in fungi. Ann. Microbiol..

[41] Chang S., Puryear J., Cairney J. (1993). A simple and efficient method for isolating RNA from pine trees. Plant. Mol. Biol. Rep..

[42] Lieberman I., Kornberg A., Simms E.S. (1955). Enzymatic synthesis of pyrimidine nucleotides; orotidine-5’-phosphate and uridine-5’-phosphate. J. Biol. Chem..

[43] Nakazawa T., Tsuzuki M., Irie T., Sakamoto M., Honda Y. (2016). Marker recycling via 5-fluoroorotic acid and 5-fluorocytosine counter-selection in the white-rot agaricomycete *Pleurotus ostreatus*. Fungal Biol..

[44] Mu D., Shi L., Ren A., Li M., Wu F., Jiang A., Zhao M. (2012). The development and application of a multiple gene co-silencing system using endogenous *URA3* as a reporter gene in *Ganoderma lucidum*. PLoS ONE.

[45] Vonk P.J., Escobar N., Wösten H.A.B., Lugones L.G., Ohm R.A. (2019). High-throughput targeted gene deletion in the model mushroom *Schizophyllum commune* using pre-assembled Cas9 ribonucleoproteins. Sci. Rep..

[46] Al Abdallah Q., Ge W., Fortwendel J.R. (2017). A simple and universal system for gene manipulation in *Aspergillus fumigatus*: In *vitro*-assembled Cas9-guide RNA ribonucleoproteins coupled with microhomology repair templates. Msphere.

[47] Foster A.J., Martin-Urdiroz M., Yan X., Wright H.S., Soanes D.M., Talbot N.J. (2018). CRISPR-Cas9 ribonucleoprotein-mediated co-editing and counterselection in the rice blast fungus. Sci. Rep..

[48] Broomfield P.L., Hargreaves J.A. (1992). A single amino-acid change in the iron-sulphur protein subunit of succinate dehydrogenase confers resistance to carboxin in *Ustilago maydis*. Curr. Genet..

[49] Irie T., Sato T., Satio K., Honda Y., Watanabe T., Kuwahara M., Enei H. (2003). Construction of a homologous selectable marker gene for *Lentinula edodes* transformation. Biosci. Biotechnol. Biochem..

[50] Honda Y., Matsuyama T., Irie T., Watanabe T., Kuwahara M. (2000). Carboxin resistance transformation of the homobasidiomycete fungus *Pleurotus ostreatus*. Curr. Genet..

[51] Nødvig C.S., Hoof J.B., Kogle M.E., Jarczynska Z.D., Lehmbeck J., Klitgaard D.K., Mortensen U.H. (2018). Efficient oligo nucleotide mediated CRISPR-Cas9 gene editing in Aspergilli. Fungal Genet. Biol..

[52] Agudelo D., Duringer A., Bozoyan L., Huard C.C., Carter S., Loehr J., Synodinou D., Drouin M., Salsman J., Dellaire G. (2017). Marker-free coselection for CRISPR-driven genome editing in human cells. Nat. Methods.

[53] Todokoro T., Bando H., Kotaka A., Tsutsumi H., Hata Y., Ishida H. (2020). Identification of a novel pyrithiamine resistance marker gene *thiI* for genome co-editing in *Aspergillus oryzae*. J. Biosci. Bioeng..

[54] Casado López S., Peng M., Issak T.Y., Daly P., de Vries R.P., Mäkelä M.R. (2018). Induction of genes encoding plant cell wall-degrading carbohydrate-active enzymes by lignocellulose-derived monosaccharides and cellobiose in the white-rot fungus *Dichomitus squalens*. Appl. Environ. Microbiol..

[55] Daly P., Casado López S., Peng M., Lancefield C.S., Purvine S.O., Kim Y.M., Zink E.M., Dohnalkova A., Singan V.R., Lipzen A. (2018). *Dichomitus squalens* partially tailors its molecular responses to the composition of solid wood. Environ. Microbiol..

