# Supplementary material for: Application of CRISPR/Cas9 Tools for Genome Editing in the White-Rot Fungus Dichomitus squalens
Source: Biomolecules. 2021 Oct 15;11(10):1526. doi: 10.3390/biom11101526 (PMC8533725; doi:10.3390/biom11101526)
Supplement: Supplementary file 1 [file biomolecules-11-01526-s001.zip › biomolecules-1382778-supplementary.pdf]

Supplementary Materials to the manuscript **"Application of CRISPR/Cas9 tools for genome editing in the white-rot fungus *Dichomitus squalens*"** by Kowalczyk et al.

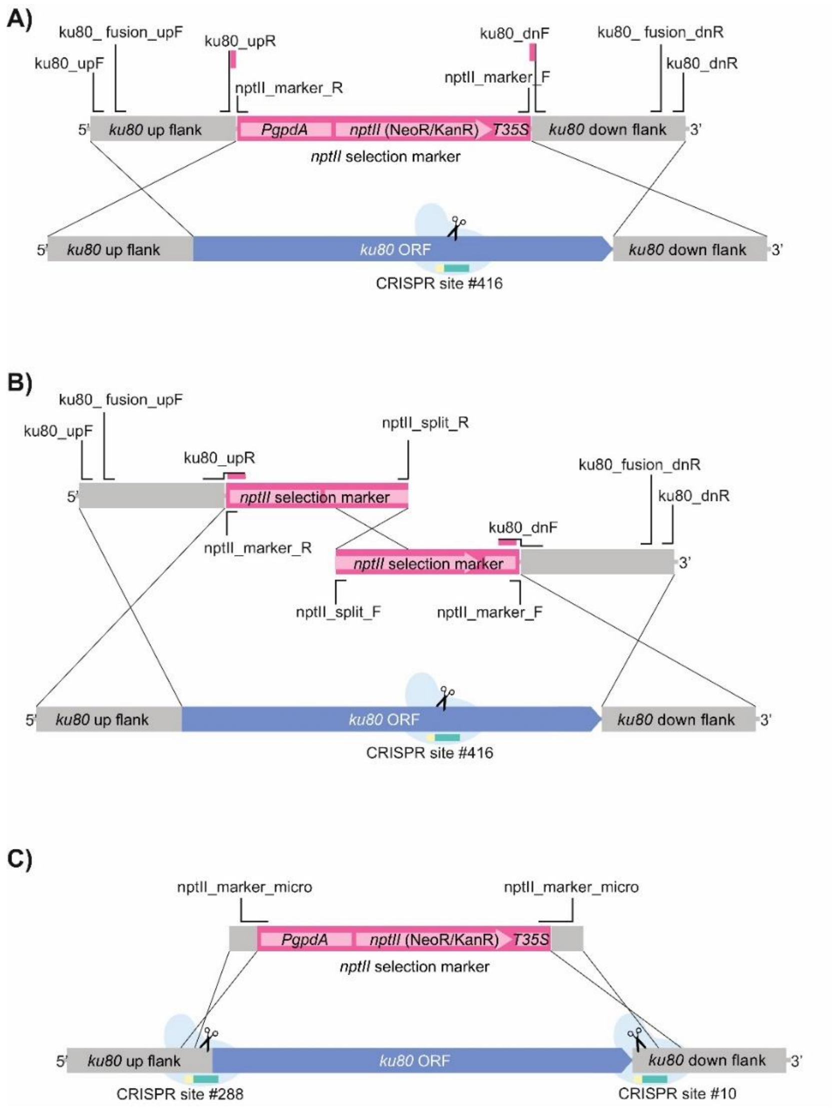

Figure S1. Construction of HDR-based donor DNA for repair of DNA breaks introduced by gRNA/Cas9 RNPs at *ku80* locus of *Dichomitus squalens*. (**a**) The linear *ku80* deletion cassette harboring geneticin resistance gene (*nptII*) and ~1.6 kb flanks homologous to *ku80* up- and down-stream regions. (**b**) The split marker deletion cassette requires an additional recombination step between the overlapping parts of the marker. (**c**) The microhomology repair template with geneticin resistance gene (*nptII*) flanked by ~40 bp homologous *ku80* tails.

(a)

```
              START
ura3 WT       ATGTCGTCTGTATCCAAGCAGACTTACGCGCAAAGGGCTGCCCAGCACCCAAACCCTGCT      60
ura3^MUT_A    ATGTCGTCTGTATCCAAGCAGACTTACGCGCAAAGGGCTGCCCAGCACCCAAACCCTGCT      60
              ************************************************************
                                                  PAM  CRISPR site #137
ura3 WT       GCAAAGGCGCTCCTCGAGACGATCGAACGCAAACGCACCAACCTCTCCGTTAGTGTGGAT      120
ura3^MUT_A    GCAAAGGCGCTCCTCGAGACGATCGAACGCAAACGCACCAACCTCTCCGTTAGTGTGGAT      120
              ************************************************************

ura3 WT       GTCACCAAACAAGAGGATTTCTTCAGGATTATCGACACCGTCGGACCGTACGTATGTCTG      180
ura3^MUT_A    GTCACCAAACAAGAGGATTTCTTCAGGATTATCGACACCGTCGGACCGTACGTATGTCTG      180
              ************************************************************

ura3 WT       TTGAAGGTAGGTGATCGAGTTTTTGCGAATATTCACGAAAGGCTAACCGATGCCATGAAA      240
ura3^MUT_A    TTGAAGGTAGGTGATCGAGTTTTTGCGAATATTCACGAAAGGCTAACCGATGCCATGAAA      240
              ************************************************************

ura3 WT       GACACACGTAGATATCATTGAGGACTTCGAGCCGTCCGTGATCGAGCGCCTCAAGGAACT      300
ura3^MUT_A    GACACACGTAGATATCATTGAGGACTTCGAGCCGTCCGTGATCGAGCGCCTCAAGGAACT      300
              ************************************************************

ura3 WT       TAGCCAAAAGCATGACTTCCTGATCTTCGAGGACAGGAAGTTCGCAGACATCGGTGAGGT      360
ura3^MUT_A    TAGCCAAAAGCATGACTTCCTGATCTTCGAGGACAGGAAGTTCGCAGACATCGGTGAGGT      360
              ************************************************************

ura3 WT       CCTTCCCACATAATATCTCTGCTATACTGAGAAAAGGTAGGAAATACCGTGGCCCTACAG      420
ura3^MUT_A    CCTTCCCACATAATATCTCTGCTATACTGAGAAAAGGTAGGAAATACCGTGGCCCTACAG      420
              ************************************************************

ura3 WT       TACTCCGCAGGCGTTCACAAGATCGCGAGTTGGTCGCATATAACCAACGCTCATCCAGTC      480
ura3^MUT_A    TACTCCGCAGGCGTTCACAAGATCGCGAGTTGGTCGCATATAACCAACGCTCATCCAGTC      480
              ************************************************************
                                          PAM   CRISP site #119
ura3 WT       CCCGGACCGTCCATCATCTCTGGCCTCAAATCCGTCGGACTTCCACTCGGGAGAGGGCTC      540
ura3^MUT_A    CCCGGACCGTCCATCATCTCTGG-------------------------------------      503
              ***********************               n.504-565del

ura3 WT       CTGCTGCTCGCTGAGATGAGCACAAAAGGAAGCCTGGCTACCGGAGTGTACACGGAGGAG      600
ura3^MUT_A    -------------------------AAGGAAGCCTGGCTACCGGAGTGTACACGGAGGAG      538
                                       ***********************************

ura3 WT       GCGGTTCGAATGGCTCGGGCCAATCGCGACTTCGTGATCGGCTTCATCGCCCAGCGACGG      660
ura3^MUT_A    GCGGTTCGAATGGCTCGGGCCAATCGCGACTTCGTGATCGGCTTCATCGCCCAGCGACGG      598
              ************************************************************

ura3 WT       ATGGACGGCGTCGGCTTACGGGAAGGCGAGTCCACGCCAGACGAGGACTTACTCATTCTT      720
ura3^MUT_A    ATGGACGGCGTCGGCTTACGGGAAGGCGAGTCCACGCCAGACGAGGACTTACTCATTCTT      658
              ************************************************************

ura3 WT       ACTCCGGGCGTGGGGCTGGACACAAGGGGAGACGGCATGGGCCAGCAATATCGGACGCCC      780
ura3^MUT_A    ACTCCGGGCGTGGGGCTGGACACAAGGGGAGACGGCATGGGCCAGCAATATCGGACGCCC      718
              ************************************************************

ura3 WT       AGGGAGGTAATCGTAGAGTCCGGCTGCGACGTCATCATCGTTGGTCGAGGGATTTACGGG      840
ura3^MUT_A    AGGGAGGTAATCGTAGAGTCCGGCTGCGACGTCATCATCGTTGGTCGAGGGATTTACGGG      778
              ************************************************************

ura3 WT       AAAGACAATGGTGCCAATGTCGAGGCTGTCCGAGCACAAGCGGAGAGGTATCGTGCAGAG      900
ura3^MUT_A    AAAGACAATGGTGCCAATGTCGAGGCTGTCCGAGCACAAGCGGAGAGGTATCGTGCAGAG      838
              ************************************************************
                                                      STOP
ura3 WT       GGATGGAAAGCATATCGGGAGAGGGTCGATATCTCAGACTGA  942
ura3^MUT_A    GGATGGAAAGCATATCGGGAGAGGGTCGATATCTCAGACTGA  880
              ******************************************
```

(b)

```
              START
ura3 WT       ATGTCGTCTGTATCCAAGCAGACTTACGCGCAAAGGGCTGCCCAGCACCCAAACCCTGCT      60
ura3^MUT_B    ATGTCGTCTGTATCCAAGCAGACTTACGCGCAAAGGGCTGCCCAGCACCCAAACCCTGCT      60
              ************************************************************
                                                  PAM  CRISPR site #137
ura3 WT       GCAAAGGCGCTCCTCGAGACGATCGAACGCAAACGCACCAACCTCTCCGTTAGTGTGGAT      120
ura3^MUT_B    GCAAAGGCGCTCCTCGAGACGATCGAACGCAAACGCACCAAC------------------      102
              ******************************************    n.103-517del

ura3 WT       GTCACCAAACAAGAGGATTTCTTCAGGATTATCGACACCGTCGGACCGTACGTATGTCTG      180
ura3^MUT_B    ------------------------------------------------------------

ura3 WT       TTGAAGGTAGGTGATCGAGTTTTTGCGAATATTCACGAAAGGCTAACCGATGCCATGAAA      240
ura3^MUT_B    ------------------------------------------------------------

ura3 WT       GACACACGTAGATATCATTGAGGACTTCGAGCCGTCCGTGATCGAGCGCCTCAAGGAACT      300
ura3^MUT_B    ------------------------------------------------------------
```

```
ura3 WT      TAGCCAAAAGCATGACTTCCTGATCTTCGAGGACAGGAAGTTCGCAGACATCGGTGAGGT      360
ura3MUT_B    ------------------------------------------------------------

ura3 WT      CCTTCCCACATAATATCTCTGCTATACTGAGAAAAGGTAGGAAATACCGTGGCCCTACAG      420
ura3MUT_B    ------------------------------------------------------------

ura3 WT      TACTCCGCAGGCGTTCACAAGATCGCGAGTTGGTCGCATATAACCAACGCTCATCCAGTC      480
ura3MUT_B    ------------------------------------------------------------

                                            PAM   CRISP site #119
ura3 WT      CCCGGACCGTCCATCATCTCTGGCCTCAAATCCGTCGGACTTCCACTCGGGAGAGGGCTC      540
ura3MUT_B    -------------------------------------GACTTCCACTCGGGAGAGGGCTC      125
                                                  ***********************

ura3 WT      CTGCTGCTCGCTGAGATGAGCACAAAAGGAAGCCTGGCTACCGGAGTGTACACGGAGGAG      600
ura3MUT_B    CTGCTGCTCGCTGAGATGAGCACAAAAGGAAGCCTGGCTACCGGAGTGTACACGGAGGAG      185
             ************************************************************

ura3 WT      GCGGTTCGAATGGCTCGGGCCAATCGCGACTTCGTGATCGGCTTCATCGCCCAGCGACGG      660
ura3MUT_B    GCGGTTCGAATGGCTCGGGCCAATCGCGACTTCGTGATCGGCTTCATCGCCCAGCGACGG      245
             ************************************************************

ura3 WT      ATGGACGGCGTCGGCTTACGGGAAGGCGAGTCCACGCCAGACGAGGACTTACTCATTCTT      720
ura3MUT_B    ATGGACGGCGTCGGCTTACGGGAAGGCGAGTCCACGCCAGACGAGGACTTACTCATTCTT      305
             ************************************************************

ura3 WT      ACTCCGGGCGTGGGGCTGGACACAAGGGGAGACGGCATGGGCCAGCAATATCGGACGCCC      780
ura3MUT_B    ACTCCGGGCGTGGGGCTGGACACAAGGGGAGACGGCATGGGCCAGCAATATCGGACGCCC      365
             ************************************************************

ura3 WT      AGGGAGGTAATCGTAGAGTCCGGCTGCGACGTCATCATCGTTGGTCGAGGGATTTACGGG      840
ura3MUT_B    AGGGAGGTAATCGTAGAGTCCGGCTGCGACGTCATCATCGTTGGTCGAGGGATTTACGGG      425
             ************************************************************

ura3 WT      AAAGACAATGGTGCCAATGTCGAGGCTGTCCGAGCACAAGCGGAGAGGTATCGTGCAGAG      900
ura3MUT_B    AAAGACAATGGTGCCAATGTCGAGGCTGTCCGAGCACAAGCGGAGAGGTATCGTGCAGAG      485
             ************************************************************

                                                     STOP
ura3 WT      GGATGGAAAGCATATCGGGAGAGGGTCGATATCTCAGACTGA  942
ura3MUT_B    GGATGGAAAGCATATCGGGAGAGGGTCGATATCTCAGACTGA  527
             ******************************************
```

(c)

```
915108       CAGGAGCGGAGGAGGGAGACCAAGTGACCCTCGTGACGGCGAGCGTGAGGTTGAGACGAA      60
ura3MUT_B    CAGGAGCGGAGGAGGGAGACCAAGTGACCCTCGTGACGGCGAGCGTGAGGTTGAGACGAA      60
             ************************************************************

915108       TCCGCTTTGAATCTACAGAGCCCGTGGTTGAGACATTCTGTATGCGGCGCATGCCTGCTG      120
ura3MUT_B    TCCGCTTTGAATCTACAGAGCCCGTGGTTGAGACATTCTGTATGCGGCGCATGCCTGCTG      120
             ************************************************************

915108       CGCGGACCCGATCGCCCTGCGAAGAGTGAAGTCAGCATGAGGCAATGCGTATGATAGACC      180
ura3MUT_B    CGCGGACCCGATCGCCCTGCGAAGAGTGAAGTCAGCATGAGGCAATGCGTATGATAGACC      180
             ************************************************************

915108       GAATGGGCGTGCCTCCTGAATAATATTATACAGATGCCACATGTCTTCATCATCCTCGGG      240
ura3MUT_B    GAATGGGCGTGCCTCCTGAATAATATTATACAGATGCCACATGTCTTCATCATCCTCGGG      240
             ************************************************************

915108       GCGGACAGTGACACGACCCTGAGTGTAGACATGCGTTAGTTGAGCGACCCCGC             294
ura3MUT_B    GCGGACAGTGACACGACCCTGAGTGTAGACATGCGTTAGTTGAGCGACCCCGC             294
             *****************************************************

protein ID: 915108
```

Figure S2. Sanger sequencing of *D. squalens ura3*$^{MUT\_A}$ and *ura3*$^{MUT\_B}$ strains. (**a**) The *D. squalens ura3*$^{MUT\_A}$ has a 61 bp deletion, n.103-517del, near CRISPR site #119 in comparison to *ura3* gene the wild type *D. squalens* strain (WT). (**b**) The *D. squalens ura3*$^{MUT\_B}$ has a 414 bp long deletion, n.103-517del, between CRISPR sites #137 and #119, (**c**) and a 294 bp long insertion that was identified to be a fragment of a putative meiotic cell division protein with protein ID: 915108.

Table S1. Sequences of CRISPR/Cas9 guides.

Target	CRISPR/Cas9 site ID	Sequence (5' → 3')	PAM	Location	Activity Score (Doench et al. [38])	Specificity Score (Hsu et al. [39])	#Off-target Sites
ura3	CRISPR guide #137	GACATCCACACTAACGGAG	AGG	1st exon	0.756	100.00%	0 (0 in CDS)
	CRISPR guide #130	CTTTTGGCTAAGTTCCTTG	AGG	2nd exon	0.672	100.00%	0 (0 in CDS)
	CRISPR guide #119	CTCCCGAGTGGAAGTCCGA	CGG	3rd exon	0.656	100.00%	0 (0 in CDS)
sdi1	CRISPR guide #40	ATTGAAGATCGTGTGGCAA	CGG	exon	0.464	99.91%	1 (0 in CDS)
	CRISPR guide #71	ATTCTGCATCTTCTCCTTA	CGG	exon	0.446	100.00%	0 (0 in CDS)
	CRISPR guide #29	AAAGCTCCACAACGAGCGT	GGG	intron	0.402	99.62%	1 (0 in CDS)
ku80	CRISPR guide #376	CTCGCCGCCGGACGATGCT	GGG	1st exon	0.208	100.00%	0 (0 in CDS)
	CRISPR guide #382	TGATCAACGACGCTAACGG	TGG	2nd exon	0.496	100.00%	0 (0 in CDS)
	CRISPR guide #386	AGACCCAAGACCAATACCT	CGG	3rd exon	0.761	100.00%	0 (0 in CDS)
	CRISPR guide #416	CAAATACATGGACCCCCCG	CGG	8th exon	0.899	100.00%	0 (0 in CDS)
	CRISPR guide #288	TGCTACTACCCCTTCGACA	CGG	*ku80* 5′ flank	0.024	100.00%	0 (0 in CDS)
	CRISPR guide #10	CAGAATTGATCAAGGACGG	AGG	*ku80* 3′ flank	0.116	88.59%	14 (10 in CDS)
lcc3	CRISPR guide #270	GAAGACCACTAGTATCGTA	CGG	2nd exon/ intron	0.581	100.00%	0 (0 in CDS)
mnp2	CRISPR guide #241	AGTTCGTGCCCGTGAGTCG	CGG	1st exon/ intron	0.675	100.00%	0 (0 in CDS)

Table S2. PCR primers used for guide RNA synthesis.

Primer name	Sequence (5' → 3')
ura3 guide #137 F	TAATACGACTCACTATAGGACATCCACACTAACGGAG
ura3 guide #137 R	TTCTAGCTCTAAAACCTCCGTTAGTGTGGATGTC
ura3 guide #130 F	TAATACGACTCACTATAGCTTTTGGCTAAGTTCCTTG
ura3 guide #130 R	TTCTAGCTCTAAAACCAAGGAACTTAGCCAAAAG
ura3 guide #119 F	TAATACGACTCACTATAGCTCCCGAGTGGAAGTCCGA
ura3 guide #119 R	TTCTAGCTCTAAAACTCGGACTTCCACTCGGGAG
sdi1 guide #40 F	TAATACGACTCACTATAGATTGAAGATCGTGTGG
sdi1 guide #40 R	TTCTAGCTCTAAAACTTGCCACACGATCTTCAAT
sdi1 guide #71 F	TAATACGACTCACTATAGATTCTGCATCTTCTCC
sdi1 guide #71 R	TTCTAGCTCTAAAACTAAGGAGAAGATGCAGAAT
sdi1 guide #29 F	TAATACGACTCACTATAGAAAGCTCCACAACGAG
sdi1 guide #29 R	TTCTAGCTCTAAAACACGCTCGTTGTGGAGCTTT
ku80 guide #376 F	TAATACGACTCACTATAGCTCGCCGCCGGACGATGCT
ku80 guide #376 R	TTCTAGCTCTAAAACAGCATCGTCCGGCGGCGAG
ku80 guide #382 F	TAATACGACTCACTATAGTGATCAACGACGCTAACGG
ku80 guide #382 R	TTCTAGCTCTAAAACCCGTTAGCGTCGTTGATCA
ku80 guide #386 F	TAATACGACTCACTATAGAGACCCAAGACCAATACCT
ku80 guide #386 R	TTCTAGCTCTAAAACAGGTATTGGTCTTGGGTCT
ku80 guide #416 F	TAATACGACTCACTATAGCAAATACATGGACCCCCCG
ku80 guide #416 F	TTCTAGCTCTAAAACCGGGGGGTCCATGTATTTG
ku80 guide #288 F	TAATACGACTCACTATAGTGCTACTACCCCTTCGACA
ku80 guide #288 R	TTCTAGCTCTAAAACTGTCGAAGGGGTAGTAGCA
ku80 guide #10 F	TAATACGACTCACTATAGCAGAATTGATCAAGGACGG
ku80 guide #10 R	TTCTAGCTCTAAAACCCGTCCTTGATCAATTCTG
Ds_lcc3 guide #270 F	TAATACGACTCACTATAGGAAGACCACTAGTATCGTA
Ds_lcc3 guide #270 R	TTCTAGCTCTAAAACTACGATACTAGTGGTCTTC
Ds_mnp2 guide #241 F	TAATACGACTCACTATAGAGTTCGTGCCCGTGAGTCG
Ds_mnp2 guide #241 R	TTCTAGCTCTAAAACCGACTCACGGGCACGAACT

Table S3. Primers used to construct homology-based repair temples for *ku80* deletion. The assembly schema is visualized in Figure S1.

Primer name	Description	Sequence 5'-3'	Amplicon size (bp)
A) For linear and split marker repair templates			
nptII_marker_F	Amplifies outside T35S terminator in pFungiway8	GATGGGCTGCCTGTATCGAG	1993
nptII_marker_R	Amplifies outside P*gpdA* in pFungiway8	GCCGCTCTAGACTCTTGGC	
ku80_upF	Amplifies *ku80* upstream flank	CGCTCAGCAACGTCTCAATC	1638
ku80_upR	Amplifies *ku80* upstream flank with complementary *nptII* marker overhang	TCGTCCAGTACCAGCCAAGAGTCTAGAGCGTGACTTAGCATACGAACAGCATCG	
ku80_dnF	Amplifies *ku80* downstream flank with complementary *nptII* marker overhang	AAAATCACCACTCGATACAGGCAGCCCATCGACATTCGGCATCTAACTCTGC	1667
ku80_dnR	Amplifies *ku80* downstream flank	CGTCAACTGCTGTGATCTGC	
ku80_ fusion_upF	Nested primer for *ku80* flank and *nptII* marker fusion	CCTCTAGTCTCGATGGACAGC	5147
ku80_ fusion_dnR	Nested primer for *ku80* flank and *nptII* marker fusion	GCAGGTGAGTCCTTCATCCC	
nptII_split_F	Amplifies split marker template with ku80_ fusion_dnR	GGTGCCCTGAATGAACTCCA	2648
nptII_split_R	Amplifies split marker template with ku80_ fusion_upF	AAAAGCGGCCATTTTCCACC	2947
B) For microhomology repair template			
nptII_marker_micro	Amplifies *nptII* marker with 38 nt tail homologous to *ku80* up-flanking region	GGCACCGCGACAAGCCGCGCCTCCTCACGTTGCTACTACGCTCTAGACTCTTGGCTGGTA	1993
nptII_marker_micro R	Amplifies *nptII* marker with 40 nt tail homologous to *ku80* dn-flanking region	GACGATGGTCTGCGCGTTCGCGGAATGCGCAGCAGAATTGGATGGGCTGCCTGTATCGAG	

Table S4. Sequences of ssODNs.

Oligo name	Sequence
ssODN #1 sdi1 120 bp	GACACCTACAAGGCGCATCGTAAGGAGAAGATGCAGAATGAGCTCAGTCTCTAT CGTTGCCTCACGATCTTCAACTGCGCGCGCACGTGCCCCAAGGGCCTGAACCCCG CCGCTGCCATC
ssODN #2 sdi1 100 bp	CAAGGCGCATCGTAAGGAGAAGATGCAGAATGAGCTCAGTCTCTATCGTTGCCTC ACGATCTTCAACTGCGCGCGCACGTGCCCCAAGGGCCTGAACCCC
ssODN #3 sdi1 80 bp	CGCATCGTAAGGAGAAGATGCAGAATGAGCTCAGTCTCTATCGTTGCCTCACGAT CTTCAACTGCGCGCGCACGTGCCCC
ssODN #4 sdi1 60 bp	GTAAGGAGAAGATGCAGAATGAGCTCAGTCTCTATCGTTGCCTCACGATCTTCAA CTGCG
ssODN #5 sdi1 40 bp	GTCTCTATCGTTGCCTCACGATCTTCAACTGCGCGCGCAC
ssODN #6 sdi1 120 bp	CGTCTCACGCTCGTTGTGGATCCTTGGCAGGCCTAATGCGACACGCGCATACAGG ACACCTACAAGGCGCATCGTAAGGAGAAGATGCAGAATGAGCTCAGTCTCTATC GTTGCCTCACG
ssODN #7 ku80 stop	ATGTTCCTCGTCGACATCTAGCCTTCCATGGGCAAGATGCGAGAATTCGAGGTCTC AGCATAGTCCGGCGGCGAGTCCGAGGTCATCGAGATGACCAACCTCGAGTGGTCA CTGCAGTTC
ssODN #8 ku80 stop	GACCGACAAGTGTGGCGTCATTCTCTTCGGTAGCGAAGAGACGAACAACATGATC AACGACGCTAACGGATCCTACGAACATGTCGCGGAGTAAATCCCCATCGCCCAGC CCAACTCGGC
ssODN #9 ku80 stop	AGACTGACTTCTGCCCGCAGCCATCGACGCTCTTATTGTAGCTATCGAGACCCAAG ACCAATAACTCAGCAACAAGAAGACGTGAACACGCAAAGTTGTCATATGGACCG ACGGCGAGAG
ssODN #19 lcc3 stop	AGGGCGACCGCTTCCAGCTTAATGTTGTCGACTAGTTGACCAACCACACTATGTTG TAGACCACTAGTATCGAATTCCCTATGCTCCGCCTACTACCTCATATGTTGTGCTA ACAACAGT
ssODN #17 mnp2 stop	GCCCCGAGCCGCCAGACGGTGTGCTCGGACGGCACCGTCGTCCCCGACTCGGTGT GATGCGAATTCGTGCCCGTGAGTCGCGGTTGCGCTGCGCTTACCCCCGACGCCGA GCACTCACGT

Table S5. Primers used for verification of edited loci.

Primer name	Sequence (5' → 3')	Amplicon size (bp)
ura3_seq_Fw	GACATGGTCGGGAGCTAGAG	1261
ura3_seq_Rv	AAGCGAAGGAGGAGGAAGAG	
sdi1_seq_Fw	AACATCAACGGCCAGAACAC	989
sdi1_seq_Rv	ATCCCGTCAGGCAGTACAAG	
ku80_seq_Fw	CATCGCCTCTTGCTGACTC	1002
ku80_seq_Rv	TAGTGTGAGTGCGGTGTTGC	
lcc3_seq_Fw	CCTTTGGTCTTCGAGCAGTC	990
lcc3_seq_Rv	TACCGCTTGCCCTGAGTAAC	
mnp2_seq_Fw	CACATCCTTGTGAACGCAAC	957
mnp2_seq_Rv	TCTGAAAGGAGCAGGGAGTG	
